# A 5G smartphone-oriented dual-band dual-antenna system designed via characteristic mode theory and surface current analysis

**DOI:** 10.1038/s41598-025-12308-9

**Published:** 2025-07-30

**Authors:** Zhaozhi Gu, Wenhan Li, Shibao Li

**Affiliations:** School of Oceanography and Space Informatics, China University of Petroleum (East China), Qingdao, 266580 China

**Keywords:** 5G MIMO, Dual-antenna system, Characteristic mode theory, Surface current distribution, Neutralization line (NL), Defected ground structure (DGS), Engineering, Electrical and electronic engineering

## Abstract

In this research, a compact dual-band dual-antenna system with mutual coupling reduction based on characteristic mode theory and surface current distribution for 5G mobile terminals is proposed. The edge-to-edge distance between two antennas is only 5 mm. The dimensions of the antenna system are 6 mm × 34 mm (approximately 0.07λmin × 0.41λmin, where λmin corresponds to the lowest operational frequency). The design comprises two modified inverted-F antenna elements with parasitic feeding, which are arranged in a back-to-back configuration. A defected ground structure (DGS) and a neutralization line (NL) are employed for decoupling in the lower (3.3–3.6 GHz) and upper (5.1–5.9 GHz) bands, respectively, achieving isolation levels of |S₂₁|< –25.5 dB and < –23.3 dB. The proposed system demonstrates compact geometry, low-cost fabrication, and scalability for array applications*.* This study presents a systematic design methodology that integrates characteristic mode analysis with current distribution optimization. The proposed approach enables direct translation of current distributions into physical antenna parameters, effectively eliminating empirical trial-and-error processes while establishing a reproducible design paradigm for future antenna development.

## Introduction

Multiple input multiple output (MIMO) technology, which uses multiple antennas for parallel signal transmission, enhances channel capacity and spectral efficiency through spatial multiplexing and diversity gains, thereby playing a vital role in 5G systems^1,2^. However, with the diversification of 5G frequency bands, MIMO antenna systems are required to support multiband coverage, posing significant challenges to antenna design. Additionally, the trend toward mobile terminal miniaturization has reduced antenna spacing, which significantly increases mutual coupling between elements. Mutual coupling can significantly degrade the radiation efficiency, gain, and isolation of antennas while also leading to reduced channel capacity and increased bit error rates, severely limiting the performance of MIMO systems. Therefore, reducing the mutual coupling of multiband antennas within limited space has become a critical issue in the design of 5G mobile terminal antennas.

Various decoupling methods, such as the decoupling network^3^, loading lumped components^4^, orthogonal diversity^5^, adding parasitic strips^6^, defected ground structure^7,8^, and neutralization line (NL) technique^9–11^, have been reported in the literature. However, a single decoupling method is generally only capable of achieving single-frequency or narrowband decoupling^12–14^. The neutralization line technique was first proposed by Diallo *et al.* to decouple two planar inverted-F antennas (PIFAs), and it achieved single-band decoupling^9^. T-shaped neutralization lines were proposed in^10,11^ which actually constructed two coupling paths for decoupling different bands. Therefore, a single neutralization line that decouples a single band essentially remains. When dual-band or wideband decoupling is needed, it can be achieved by combining multiple decoupling structures. In^12^, three neutralization lines were used for wideband decoupling. Meandering resonant branches and inverted T-shaped slots were employed to decouple the upper and lower bands of symmetrical IFAs^13^. These methods improved the isolation but resulted in a large antenna size, failing to meet miniaturization requirements. In^14^ a decoupling inductor, a neutralization line and a decoupling branch are combined to reduce wideband mutual coupling. While these combined decoupling techniques have demonstrated high isolation for multi-frequency broadband antenna systems, several challenges remain. The optimization of component values and structural configurations is a complicated process. For example, it is difficult to determine the proper positions, shapes and dimensions of multiple neutralization lines because of their mutual interactions. At present, most antenna design and

decoupling structures rely on the designer’s experience and iterative trial-and-error, which requires significant time. To address these challenges, a more systematic and theoretical method is essential.

This paper proposes a dual-band dual-antenna system with decoupling methods of a combined neutralization line and a defected ground structure. First, a dual-band dipole antenna based on a modified inverted-F antenna (IFA) with coupled feeding is designed, and the dipole is then arranged to a back-to-back two-antenna array. A defected ground structure and a neutralization line are introduced for lower band and upper band decoupling, respectively. Thus, a compact and high-isolation dual-band dual-antenna system is achieved. Moreover, the design process is guided by characteristic mode theory^15–18^ and analysis of the surface current distribution, providing a rigorous theoretical paradigm. This method significantly improves design efficiency and offers valuable theoretical insights for future antenna design. The rest of this paper is organized as follows. Section “Relevant technology” details the design of the dual-band antenna element on the basis of characteristic mode theory and the working mechanism of the two decoupling structures on the basis of the analysis of the surface current distribution. The antenna is fabricated and tested in Section “Experimental Results and Discussion”, and conclusions are drawn in Section “Conclusions”.

## Relevant technology

### Fundamental theory of characteristic mode

The characteristic mode analysis (CMA) provides physical insight into the inherent resonant properties of the antenna structure. The modes are solutions to the generalized eigenvalue equation derived from the method of moments impedance matrix

$$XJ_{n} = \lambda_{n} RJ_{n}$$

where $R$ and $X$ represent the real and imaginary parts of the impedance matrix respectively, $J_{n}$ denotes the characteristic current distribution, and $\lambda_{n}$ is the eigenvalue of the n-th mode. The modal significance (MS) is derived as:

$$MS_{n} = \frac{1}{{\left| {1 + j\lambda_{n} } \right|}}$$

The modal significance quantifies how easily a mode can be excited at specific frequencies. Modes become easier to excite as their modal significance gets closer to 1. Both simulations and measurements show this threshold could potentially extend to 0.5.Notably, CMA predicted frequencies represent structural resonances, while the actual excitation frequencies observed in S-parameters are achieved through proper feed placement matching modal current distributions and careful structural parameter sweeps. The dual-band array element design in Section “Dual-frequency array element design” adopts this approach.

### Envelope correlation coefficient

In MIMO antenna systems, lower correlation indicates better diversity performance and weaker coupling between antenna elements. The Envelope Correlation Coefficient (ECC) measures this inter-antenna correlation.

It can be calculated from the antenna’s S-parameters using the following formula:

$$ECC = \frac{{\left| {S_{11}^{*} S_{12} + S_{21}^{*} S_{22} } \right|^{2} }}{{\left| {\left( {1 - \left| {S_{11} } \right|^{2} - \left| {S_{21} } \right|^{2} } \right) \cdot \left( {1 - \left| {S_{22} } \right|^{2} - \left| {S_{12} } \right|^{2} } \right)} \right|}}$$

In practical MIMO systems, the ECC between antenna elements should be below 0.5.

## Dual-band dual-antenna system and decoupling mechanisms

### Dual-band antenna system geometry

**Fig. 1:**
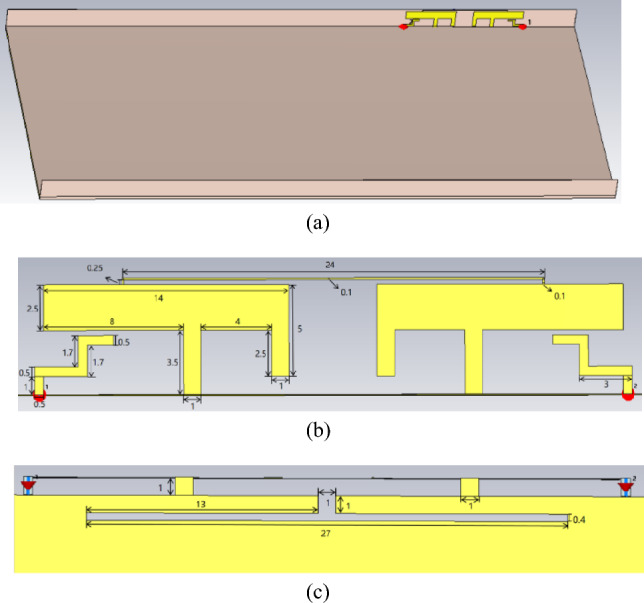
Geometry of the dual-band dual-antenna system (unit: mm).

The geometry and dimensions of the proposed dual-band dual-antenna system are shown in Fig. 1. The antenna structure consists of one FR-4 dielectric substrate with a thickness of 0.8 mm and two FR-4 dielectric substrates with a thickness of 0.1 mm (ε_r_ = 4.4, tan δ = 0.02). The bottom dielectric substrate measures 160 mm × 75 mm × 0.8 mm, whereas the side dielectric substrates measure 160 mm × 6.5 mm × 0.1 mm. The metal ground plane is printed on the opposite side of the dielectric substrate, approximately 1 mm away from the edge of the main dielectric substrate. The patch antenna is printed on the inner side of the main dielectric substrate and is composed of two back-to-back modified coupling-fed inverted-F antennas, a defected ground structure and a neutralization line for low-frequency and high-frequency decoupling, respectively. It works in the 3.3–3.6 GHz band and 5.1–5.9 GHz band.

### Dual-frequency array element design

**Fig. 2:**
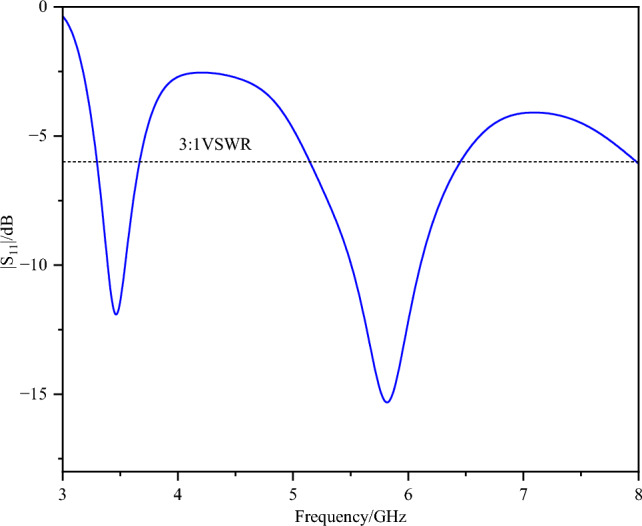
S_11_ of the array element.

**Fig. 3:**
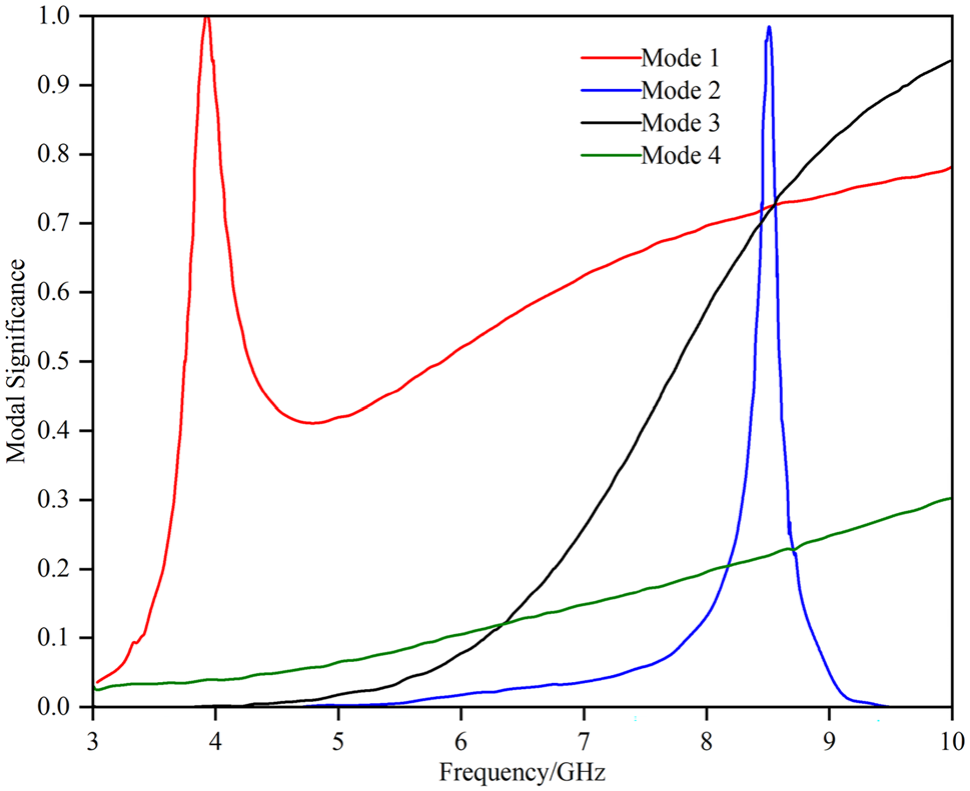
Modal significance of the initial structure.

Figure 2 shows the S₁₁ of the array element. The antenna operates in two frequency bands: 3.28–3.66 GHz and 5.10–6.45 GHz. The design process and detailed analysis based on characteristic mode theory are described below. In this design, the shorting end of the conventional inverted-F antenna is opened, while the feeding point is shorted. The initial antenna structure is analysed via characteristic mode theory, and the modal significance is illustrated in Fig. 3.

**Fig. 4:**
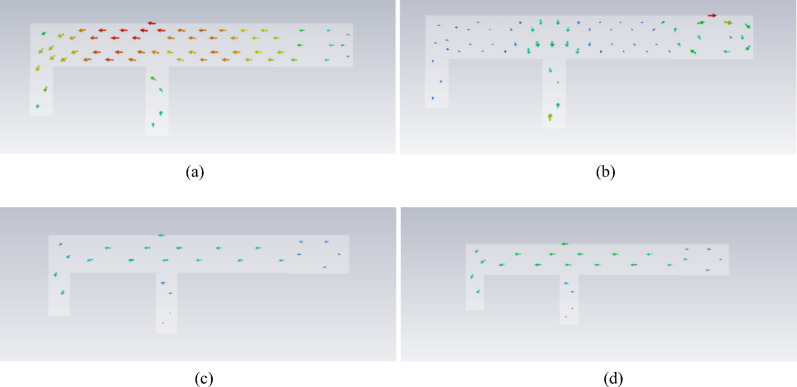
Modal current distribution.

The antenna is most easily excited at the 3.9 GHz band of Mode 1 and the 8.48 GHz frequency of Mode 2. The low-frequency band matches the target frequency range of the antenna, whereas the high-frequency band is approximately 3 GHz higher than the desired 5–6 GHz range. In the 5 GHz and 6 GHz bands, the modal significance of Mode 1 is approximately 0.5, suggesting that it can potentially be excited. The modal current distributions around these frequency bands are illustrated in Fig. 4.

To achieve excitation of the corresponding frequency points within the 5 GHz--6 GHz range, an analysis of mode significance and mode current distribution is conducted. Three potential approaches are proposed. First, in Mode 2 at 8.4 GHz, the current path forms a loop, which is primarily concentrated at the end of the antenna’s long arm and near the grounding point. By increasing the width of the antenna’s radiating arm, the current path

can be extended, thereby lowering the excitable frequency band. Second, the current distributions of Mode 1 at 5 GHz and 6 GHz are similar to that at 3.9 GHz. Consequently, exciting Mode 1 at points where the current paths for these three frequencies overlap may simultaneously stimulate the two target frequency bands. Third, the introduction of new parasitic branches could be considered to broaden the frequency band. However, the first method is disregarded because it increases the antenna size. Both the second and third methods necessitate a detailed analysis of the antenna’s feeding position; thus, they are examined in conjunction.

**Fig. 5:**
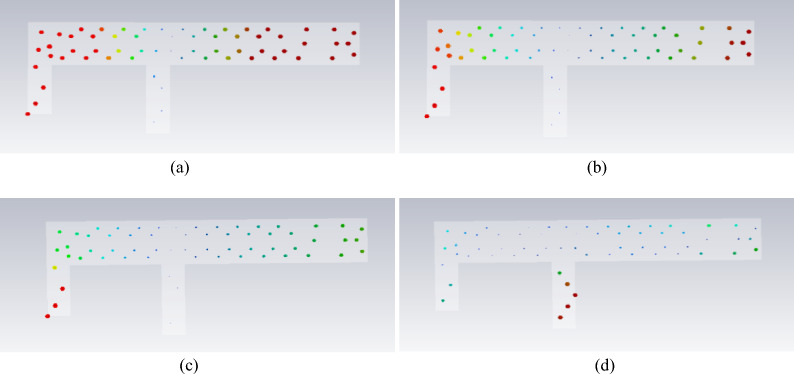
Model electric field distribution.

The electric field distributions for the modes discussed above are depicted in Fig. 5. A shared characteristic among the four frequency points is the higher electric field density observed at the terminus of the long arm.

**Fig. 6:**
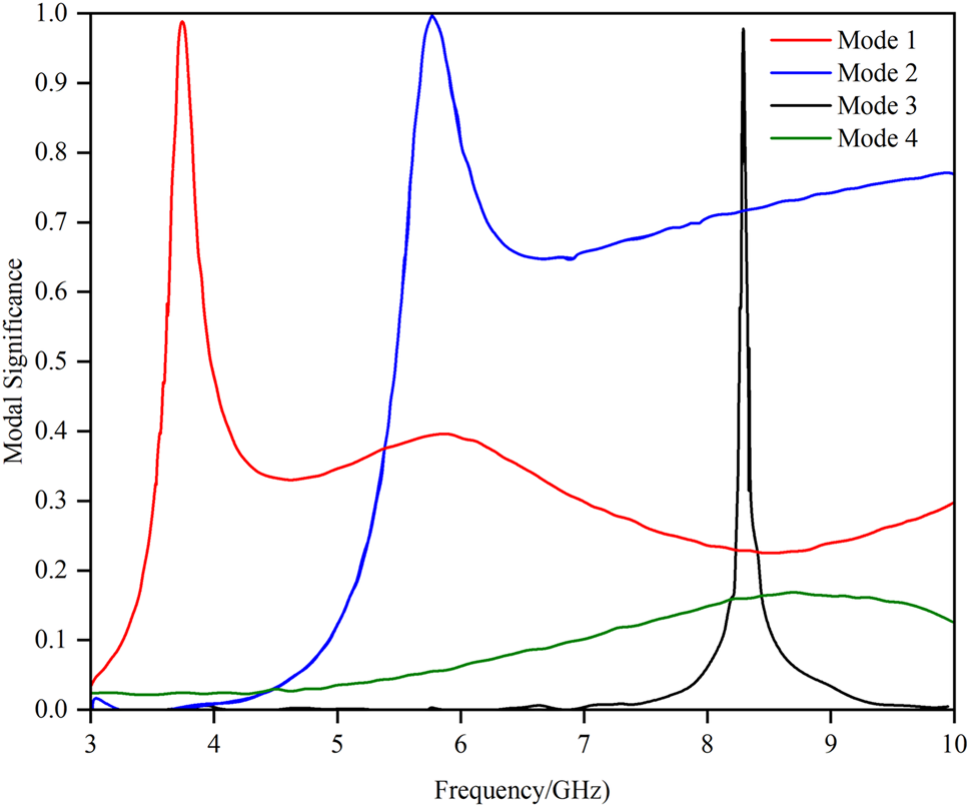
Model significance of the initial antenna design with a parasitic branch.

**Fig. 7:**
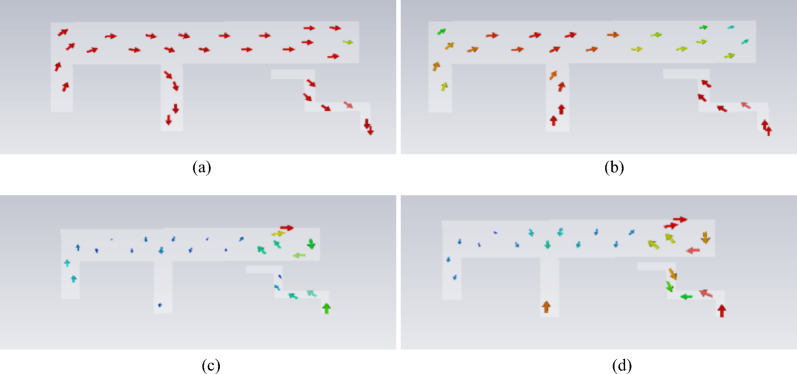
Model currents of the initial antenna design with a parasitic branch.

The feeding point is strategically placed at the region of peak electric field intensity within the mode. Considering space limitations and the need for effective excitation, a parasitic branch was added near the long arm’s end for the coupling feed. The initial antenna design with a parasitic branch feed was analysed via characteristic mode theory. The mode significance and mode current distributions are shown in Fig. 6 and Fig. 7, respectively.

It is evident that introducing a parasitic element for gap coupling yields notable improvements. First, it synchronizes the currents at 3.7 GHz and 5.7 GHz, satisfying the current path requirements for 3.9 GHz, 5 GHz, and 6 GHz, as depicted in Fig. 4. Moreover, the model current distribution suggests that higher frequencies, such as 8.3 GHz and 9.6 GHz, are also likely to be excited. The initial structure was fed on the parasitic branch, and a parameter sweep was conducted on each segment. The resulting S_11_ is illustrated in Fig. 2, showing frequency coverages of 3.28–3.66 GHz and 5.10–6.45 GHz, with a return loss reaching -16 dB at higher frequencies.

**Fig. 8:**
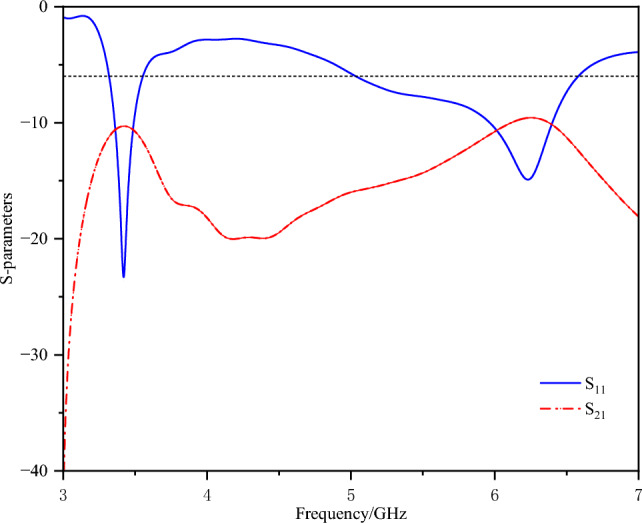
S-parameters of the two-element array without decoupling.

**Fig. 9:**
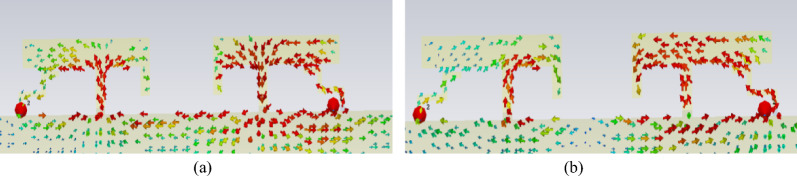
Surface currents of the two-element array without decoupling.

The designed single element was arranged into a back-to-back two-element array with a separation of 5 mm. The corresponding S-parameters are presented in Fig. 8, and the coupling current distribution is illustrated in Fig. 9 (with port 1 excited and port 2 terminated with a 50-ohm matched load). The antenna exhibits degraded impedance bandwidth performance, frequency-shifted resonant points and low isolation levels.

It reveals the presence of a coupled current between the two antenna elements, with isolation values of approximately -8 dB and -9 dB at the low-frequency band and the high-frequency band, respectively. The decoupling methods are presented below.

### Low-frequency decoupling

**Fig. 10:**
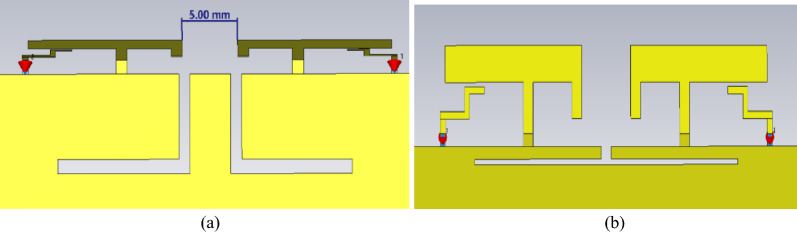
Two types of Defected Ground Structures (DGS).

The current distribution of the antennas shown in Fig. 9 reveals that low-frequency coupling is dominated by ground coupling, with the coupling path closely resembling a straight line. The high-frequency coupling is attributed to two mechanisms: ground coupling with a current null in the path and spatial wave coupling between the elements. To address these issues, two types of defected ground structures are proposed for comparison, as shown in Fig. 10.

**Fig. 11:**
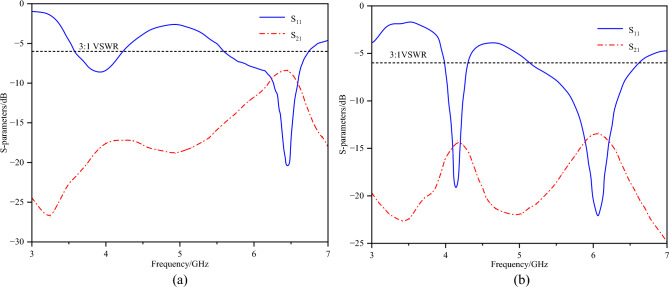
S-parameters of two types of Defected Ground Structures (DGS).

**Fig. 12:**
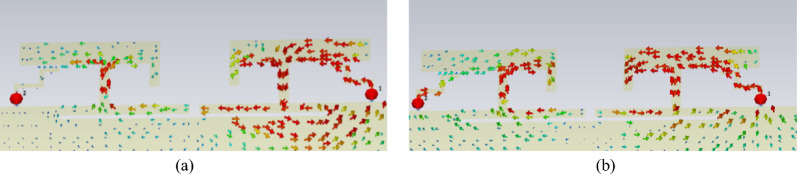
Surface current distribution with DGS.

The S-parameters of the two types of defected ground structures are shown in Fig. 11. Clearly, both slot

configurations enhance decoupling in the low-frequency range only, which is easy to understand. As discussed earlier, low-frequency coupling is driven primarily by the ground current path. Therefore, the ground slots significantly enhance decoupling at low frequencies. Considering the decoupling performance in both the 3.3-3.6GHz and 5.1-5.9GHz bands, DGS2 was chosen as the optimal structure, as demonstrated in Fig. 12.

The current distribution matches the expected results, with good isolation achieved at the low-frequency band. However, the high-frequency current distribution shows minimal change after slotting, even exhibiting slight degradation. The results exhibit both centre frequency shifts and bandwidth variations, which will be systematically corrected through optimization with the decoupling structure in the final design phase.

### High-frequency decoupling

**Fig. 13:**
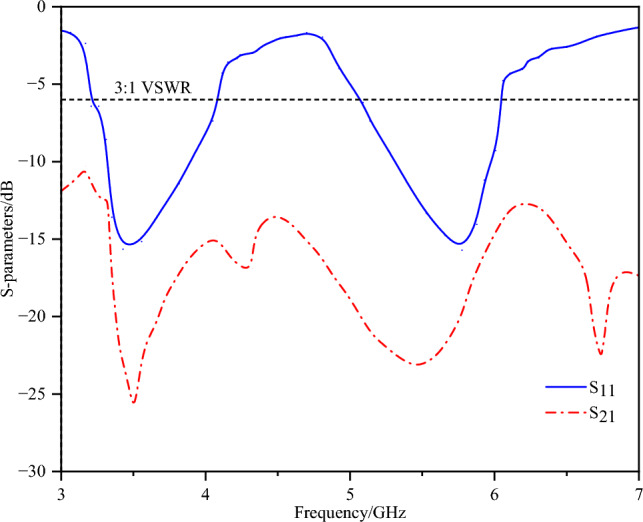
S-parameters of the dual-band dual antenna system.

The placement of the neutralization line is determined on the basis of the current distribution after loading the defected ground structure. Here, the neutralization line and the defected ground structure are not simple superposition. An analysis of the current distribution shown in Fig. 12 clearly reveals that the coupling paths for high and low frequencies differ significantly. At high frequencies, the current flows from the long side to the short side, forming a high-density current region near the short side. In contrast, at low frequencies, the current radiates outwards from the shorting point, creating a high-density current region below the long side. However, a common feature of both high- and low-frequency coupling currents is the dense current distribution along the long side. Therefore, a neutralization line connecting the long sides of the two elements is introduced, aiming to generate a reverse current flow on the line. Through estimation, a neutralization line length of approximately 15 mm is found to create a phase reversal point for high-frequency currents. Combined with parameter sweeping, the position and structural parameters of the neutralization line and the dual-antenna system are illustrated in Fig. 1. The corresponding S-parameters are shown in Fig. 13.

The dual-band dual-antenna system achieves an isolation of up to -25.5 dB at low frequencies and up to -23.3 dB at high frequencies. Since the neutralization line is placed in the common high-density current region for both high and low frequencies, it not only significantly improves high-frequency isolation but also enhances low-frequency isolation. Although a slight shift in the resonant point is observed, the performance meets the requirements across the entire operating bandwidth.

**Fig. 14:**
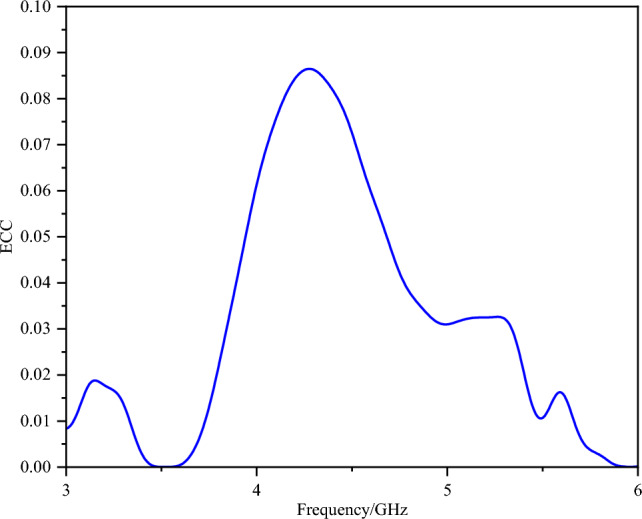
ECC of the dual-band dual-antenna system.

The envelope correlation coefficient (ECC) between antenna elements is shown in Fig. 14. Both frequency bands exhibit ECC values less than 0.05, indicating low inter-element correlation that meets standard requirements.

### Parametric study and decoupling mechanism

**Fig. 15:**
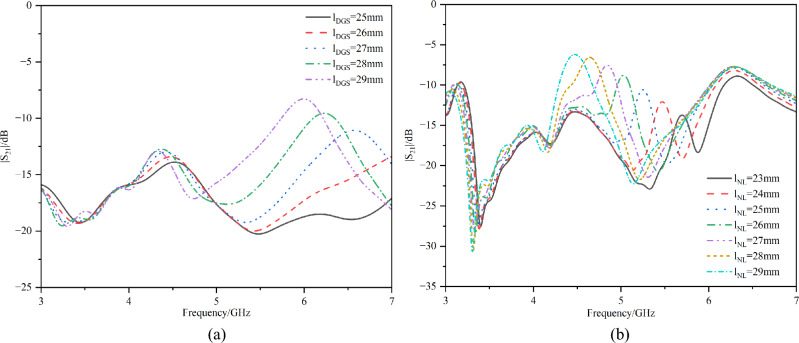
Parametric study.

To validate the design and optimize the decoupling performance, parametric studies were conducted on the Defected Ground Structure (DGS) and the Neutralization Line (NL). Figure 15 demonstrates the influence of both DGS length and the neutralization line length on the isolation characteristics (|S₂₁|) of the dual-antenna system.

It shows that as the DGS length increases, its lower-band decoupling frequency first shifts left then right, while the neutralization line’s decoupling frequency in upper band shifts left with increasing length. Final dimensions require co-optimization with the antenna’s operational band S_11_ parameters.

The decoupling mechanism, previously analysed from the perspective of current distribution, it is further investigated through transmission-line theory in this section.

At lower band, the DGS functions as a 3λ/4 open-circuited stub, where its input impedance follows:

$$Z_{in} = - jZ_{0} \cot (\frac{2\pi }{{\lambda_{g} }}l_{DGS} ) + \frac{{\lambda_{g} }}{2}$$

When $l_{DGS} \approx \frac{{3\lambda_{g} }}{4}$,$Z_{in} \to \infty$, it creates a near-ideal open circuit, blocking coupled currents between antenna elements for maximum isolation. Any length mismatch reduces impedance, weakening current blockage and degrading isolation. For the upper band (5.1–5.9 GHz), the length of the neutralization line is approximately equals to $\frac{{\lambda_{g} }}{2}$ at the centre frequency which introduces a 180° phase-inverted decoupling path, thus cancel the mutual coupling effects at upper band.

## Experimental results and discussion

**Fig. 16:**
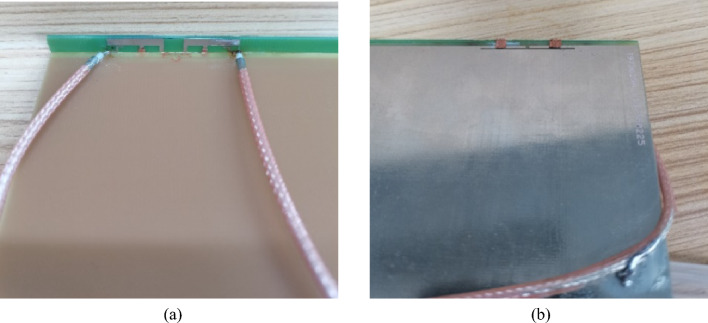
Fabricated prototype.

To experimentally validate the simulation results, a prototype according to the optimized design parameters illustrated in Fig. 1 is fabricated and tested, as shown in Fig. 16.

**Fig. 17:**
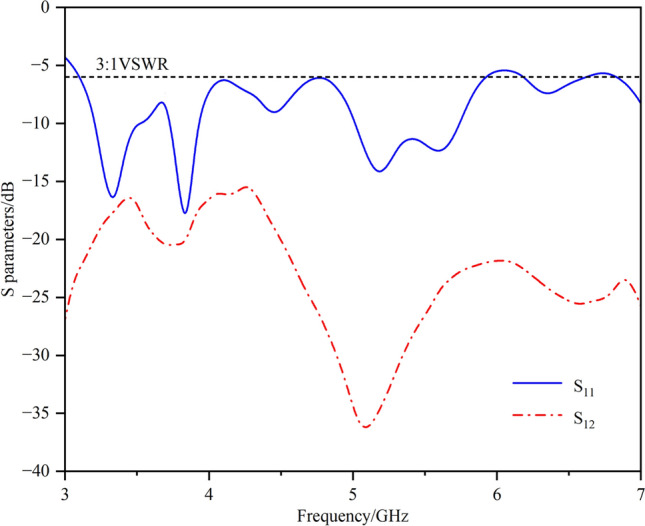
Measured S-parameters.

Figure 17 shows the measured S-parameter results. The -6 dB measured impedance bandwidth is 3.13–6.03 GHz, and the measured isolations reach -20.6 dB in the 3.3--3.6 GHz band and -30.6 dB in the 5.1--5.9 GHz band.

**Fig. 18:**
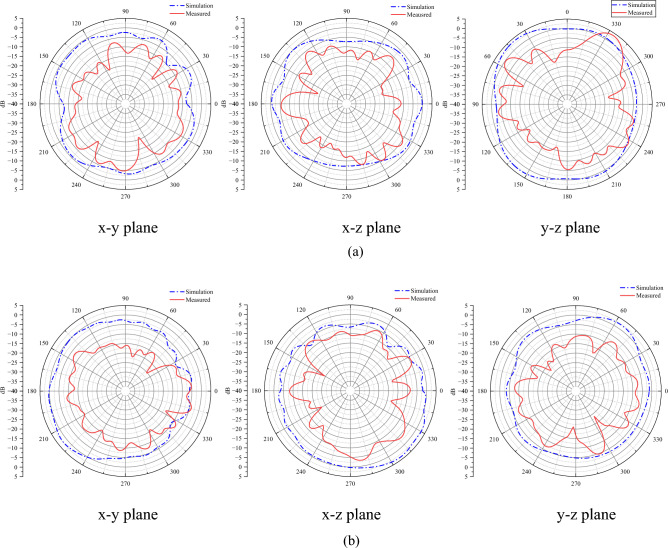
Measured far-field radiation patterns.

The measured far-field radiation patterns in x-y, x-z and y-z planes, the antenna gain and radiation efficiency are shown in Fig. 18 and Table 1, respectively. It has excellent directional characteristics, exhibiting nearly omnidirectional radiation patterns across both frequency bands. The measured efficiencies demonstrate values of -0.99dB (79.6%) at 3.5GHz and -1.1dB (77.6%) at 5.5GHz, with the peak gains are 2.23dBi and 2.17dBi, respectively.

**Table 1:** Radiation efficiency and gain of the fabricated antenna.

Frequency/GHz	Efficiency/dB	Gain/dBi
3.5	-0.99	2.23
5.5	-1.1	2.17

Table 2 exhibits a comparative evaluation of the design in this work against some existing dual-element antenna configurations. It demonstrates that the proposed dual-antenna array achieves both compact dimensions and high isolation, while its systematic design methodology effectively combines characteristic mode theory with surface current analysis.

**Table 2:** Comparison with existing dual–element designs.

Ref	Antenna size /λmin	Decoupling method	Design approach	isolation	CMA used?
^10^	1.19 × 2.99	DGS + NL	Not mentioned	< -20dB	No
^12^	1.06 × 4.16	Meandering branch + DGS	Partial current distribution	< -15dB	No
^14^	0.05 × 0.32	Lumped element + Decoupling branch + NL	Partial current distribution	< -22dB	No
This	0.07 × 0.41	DGS + NL	CMA-Current distribution	< -25dB	Yes

## Conclusions

This paper presents a compact dual-band dual-antenna system for 5G mobile terminals working at 3.3-3.6GHz and 5.1-5.9 GHz bands. The modified IFA is employed as the dual-band array element, which is designed on the basis of characteristic mode theory. The back-to-back configuration is then examined systematically. A defected ground structure and a neutralization line are employed for low-frequency and high-frequency decoupling, respectively, on the basis of the analysis of the surface current distribution. High isolation is achieved in dual bands through combined DGS and neutralization line techniques. The proposed dual-band dual-antenna system is compact and inexpensive and can be easily extended to a multi-element MIMO array system. Furthermore, this work develops a characteristic mode-guided design method that directly converts current patterns into antenna dimensions, avoiding trial-and-error optimization and offering a reliable framework for future antenna development.

## Data Availability

All antenna structural parameters, design processes and simulation data involved in this study are included in this article, thus no additional data availability statement is required. Correspondence and requests for materials should be addressed to Zhaozhi Gu.
